# Total thyroidectomy may be more reasonable as initial surgery in unilateral multifocal papillary thyroid microcarcinoma: a single-center experience

**DOI:** 10.1186/s12957-017-1130-7

**Published:** 2017-03-16

**Authors:** Shuai Xue, Peisong Wang, Jia Liu, Guang Chen

**Affiliations:** Department of Thyroid Surgery, The First Hospital of Jilin University, 130061 Changchun, People’s Republic of China

**Keywords:** Papillary thyroid microcarcinoma, Multifocality, Hemithyroidectomy, Risk factors, Recurrence

## Abstract

**Background:**

The extent of surgery in patients with unilateral multifocal papillary thyroid microcarcinoma (UMPTMC) remains to be controversial. Aimed to improve surgical management of UMPTMC, this study was performed to identify the recurrence of UMPTMC and analyze its predictive factors.

**Methods:**

This study was approved by the Ethical Committee of The First Hospital of the Jilin University, and written informed consent was given by participants for their clinical records to be used in this study. We retrospectively analyzed a total of 97 consecutive patients who underwent initial surgery for the treatment of UMPTMC at The First Hospital of Jilin University, between October 2005 and October 2006.

**Results:**

Altogether, 97 patients of UMPTMC have been enrolled in our study, in which 57 cases were performed with hemithyroidectomy (HT) while other 40 cases with total thyroidectomy (TT). The sum diameter of all tumors >1 cm was more frequent in HT group than in TT group (40.35 vs 20%; *p* = 0.046). Positive central lymph nodes were found more frequently in the TT patients than in the HT patients (80 vs 59.65%; *p* = 0.046). Tumor recurrence was seen more frequently in the HT cases than in the TT cases (26 vs 5%; *p* = 0.007). The disease-free survival period was significantly shorter for the HT patients than for the TT patients (*p =* 0.0059 by the log-rank test). The disease-free survival rates at 5 and 10 years were 91.23 and 73.68%, respectively, in the HT group and 100 and 92.5%, respectively, in the TT group. Univariate analysis by Cox’s proportional hazards method showed male gender, sum diameter of all tumors >1 cm, and central lymph node metastases (CLNM) to be risk factors for recurrence of HT patients. Male gender and sum diameter >1 cm were factors identified for multivariate analysis by Cox’s proportional hazards method which yielded risk ratios of 3.037 [CI 1.026–8.988; *p* = 0.045] and 5.475 [CI 1.389–21.572; *p* = 0.015] in the HT group.

**Conclusions:**

In summary, with an increased risk of recurrence, TT may be more reasonable as initial surgery in UMPTMC, especially with male gender and total tumor diameter greater than 1 cm.

**Electronic supplementary material:**

The online version of this article (doi:10.1186/s12957-017-1130-7) contains supplementary material, which is available to authorized users.

## Background

Papillary thyroid carcinoma (PTC) is the most common type of thyroid carcinoma, and its incidence has been increasing around the world due to widespread use of ultrasonography-guided fine needle aspiration cytology (FNAC) for small lesions [1–3]. These increases are due mostly to the increase in the detection of papillary carcinomas <1 cm in size. PTC measuring 10 mm or less in the greatest diameter, which is called papillary thyroid microcarcinoma (PTMC), accounts for 39% of the cases of thyroid cancer in the USA [3] and 43.1% of the cases in Korea [4].

However, the extent of surgery in patients with PTMC continues to be controversial. According to American Thyroid Association guidelines in 2015, hemithyroidectomy (HT) alone is sufficient treatment for small, unifocal, intrathyroidal carcinomas in the absence of prior head and neck irradiation, familial thyroid carcinoma, or clinical detectable cervical lymph node metastasis [5]. Total thyroidectomy (TT) is performed when the nodule is preoperatively confirmed to be malignant in bilateral lobes by FNAC [5]. Nevertheless, as the 17–87% of PTC were multifocal [6], there were different opinions on how to manage the unilateral multifocal PTMC (UMPTMC). Most surgeons prefer to proceed with TT, which would be associated with the lowest risk of locoregional recurrence. However, the patient would receive high-dose lifelong thyroid hormone replacement and take the higher risks of recurrent laryngeal nerve injury and hypoparathyroidism. Others would be inclined to persuade UMPTMC patients to accept HT with a slightly higher risk of locoregional recurrence. Based on these disputes, we find it important to study the clinical and pathologic predictors of recurrence in UMPTMC. Similar studies that have been published on this subject were rare [7–9]; moreover, most of these reports do not provide detailed follow-ups of the contralateral nodule. According to previous studies [7–9], the 10-year recurrent and mortality rate of UMPTMC with different surgery strategies are still unknown; therefore, the cost-effectiveness of two different surgeries is hard to evaluate.

To help elucidate these matters further, we developed a retrospective cohort study of both the HT and TT strategies in the management of UMPTMC in order to facilitate decision-making for patients and surgeons. Firstly, the objective of this study was to identify the recurrence and mortality of UMPTMC and then to analyze its predictive factors.

## Methods

### Patients

This study was approved by the Ethical Committee of The First Hospital of Jilin University, and written informed consent was given by participants for their clinical records to be used in this study. We retrospectively analyzed a total of 673 consecutive patients who underwent initial surgery for the treatment of thyroid carcinoma at The First Hospital of Jilin University, between October 2005 and October 2006. All patients recruited in the study met the following criteria: (a) patient information found in a hospital database and (b) patients with a postoperative pathological diagnosis of UMPTMC. (c) Patients underwent HT with unilateral prophylactic central lymph node dissection (CLND) or TT with bilateral prophylactic CLND. Patients were excluded from the study if they had pathological types of thyroid malignancies other than UMPTMC; preoperative confirmation of bilateral nodule, or obviously extrathyroidal invasion; preoperative confirmation of clinically positive (ultrasound positive or palpable positive) central and lateral lymph node; lack of a preoperative examination; and a history of neck radiotherapy, distant metastasis, and previous thyroid surgery. Finally, this study enrolled 97 patients with UMPTMC.

### Initial treatment and contralateral lobe evaluation

Clinical diagnosis was initially made by examination of ultrasound and FNAC. Ultrasonography cervical mapping was done by experienced, specially trained radiologist. For the UMPTMC, TT or hemithyroidectomy was performed according to the patients’ willingness. Before that, surgeons have explained both the risks and benefits of each kind of operation and given informed consent form to patients.

Ultrasound (US) of thyroid and neck lymph node was performed by two physicians with more than 5-year experience in thyroid US (Dr. Shuai Xue and Dr. Peisong Wang); they were blinded to the previously obtained findings. If their results were inconsistent, another physician with more than 15-year experience of thyroid US (Dr. Jia Liu) was asked for consultation.

### Clinicopathological variable

Demographic data on patient clinical features (gender, age at diagnosis), tumor histological characteristics (diameter of largest tumor, sum diameter of all tumors (according to histopathology reports), foci number, primary tumor laterality, extrathyroidal extension, TNM stage, presence of chronic thyroiditis), and central lymph node metastases (CLNM) were recorded.

### Follow-up, recurrence, and reoperation

Until 2011, TSH-suppressive hormonal therapy was applied to postoperative patients and all cases did not receive radioactive iodine therapy. Postoperative physical examinations were performed every 3–6 months for 10 years. During the period of follow-up, all patients underwent ultrasound examinations of the neck as well as thyroid function test (including thyroglobulin level). We considered suspicious lymph node as recurrence using FNAC and washout fluid. Meanwhile, when nodule with diameter larger than 5 mm newly occurred in the contralateral lobe, FNAC was performed to these patients. If the result was thyroid carcinoma, we defined it as recurrence and recommended completion reoperation at the same time. We took the contralateral lobe and central and lateral compartment into account as the local regional recurrence. No patients had distant metastasis. A follow-up at least 10 years post-surgery was achieved for 89 patients.

### Statistical analysis

To identify differences between groups for specific variables, SPSS version 16 software (SPSS Inc, Chicago, IL) was used for statistical analysis, which was performed by Pearson’s chi-square test or Student’s *t* test. Survival curves were drawn by Kaplan-Meier method and statistically analyzed by the log-rank test. To characterize UMPTMC, univariate analysis was performed by Cox’s proportional hazards method for disease-free survival. Factors analyzed were male gender, age (by year), diameter of the largest tumor (millimeters), sum diameter of all tumors, foci number, primary tumor laterality, extrathyroidal extension, TNM stage, presence of chronic thyroiditis, and CLNM. Multivariate analysis was performed by Cox’s proportional hazards method for disease-free survival and the risk factors. A *p* value <0.05 was considered statistically significant.

## Results

Altogether, 97 patients of UMPTMC have been enrolled in our study, in which 57 cases were performed with HT while other 40 cases with TT. The clinicopathological characteristics of the UMPTMC (*n* = 97) are shown in Table 1. The sum diameter of all tumors >1 cm was more frequent in HT group than in TT group (40.35 vs 20%; *p* = 0.046). Positive central lymph nodes were found more frequently in the TT patients than in the HT patients (80 vs 59.65%; *p* = 0.046). Tumor recurrence was seen more frequently in the HT cases than in the TT cases (26 vs 5%; *p* = 0.007). There were no significant differences between the two groups in terms of age, gender, extrathyroidal extension, diameter of the largest tumor, foci number, primary tumor laterality, TNM stage, presence of chronic thyroiditis, average follow-up period, and the number of disease-related death.

**Table 1:** Clinicopathological variable of UMPTMC patients

Characteristics		UMPTMC	
	HT(*n* = 57, %)	TT(*n* = 40, %)	*p value*
Age (years), mean ± SD	49 ± 10	53 ± 8	0.933
Age cohort			
<45 years	23(40.35)	17(42.5)	0.832
≥45 years	34(59.65)	23(57.5)	
Sex			
Male	12(21.05)	9(22.5)	0.865
Female	45(78.95)	31(77.5)	
ETE			
Yes	6(10.53)	10(25)	0.059
No	51(89.47)	30(75)	
Diameter of the largest tumor			
>5 mm	17(29.82)	12(30)	0.985
≤5 mm	40(70.18)	28(70)	
Sum diameter of all tumors			
>1 cm	23(40.35)	8(20)	*0.046*
≤1 cm	34(59.65)	32(80)	
Foci number			
>3	9(15.79)	7(17.5)	0.823
≤3	48(84.21)	33(82.5)	
Primary tumor laterality			
Left	27(47.37)	19(47.5)	0.990
Right	30(52.63)	21(52.5)	
TNM stage			
I and II	22(38.60)	12(30)	
III and IV	35(61.40)	28(70)	
With chronic thyroiditis			
Yes	5(8.77)	6(15)	0.167
No	52(91.23)	34(85)	
Follow-up period (months)			
Average ± SD	126 ± 5	128 ± 3	
CLNM			0.822
Yes	34(59.65)	32(80)	
No	23(40.35)	8(20)	*0.046*
Outcome			
Tumor recurrence	15/57(26)	2/40(5)	*0.007*
Contralateral lobe	14(24.56)	0(0)	
Central/lateral lymph node	1(1.44)	2(5)	
Disease-related death	2/57(3.5)	1/40(2.5)	1.000

*SD* standard deviation, *ETE* extrathyroidal extension, *CLNM* central lymph node metastases

**Fig. 1:**
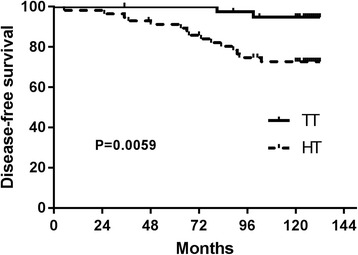
The disease-free survival rates at 5 and 10 years were 91.23 and 73.68%, respectively, in the HT group and 100 and 92.5%, respectively, in the TT group. The disease-free survival period was significantly shorter for the HT patients than for the TT patients (*p =* 0.0059 by the log-rank test)

**Fig. 2:**
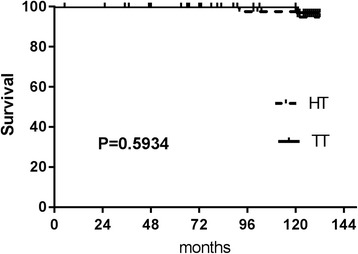
Disease-specific mortality did not differ statistically between groups

The disease-free survival period was significantly shorter for the HT patients than for the TT patients (*p =* 0.0059 by the log-rank test) (Fig. 1). Details of recurrent cases are shown in Table 2. The disease-free survival rates at 5 and 10 years were 91.23 and 73.68%, respectively, in the HT group and 100 and 92.5%, respectively, in the TT group. Survival curves did not differ statistically between groups (data shown in Table 1 and Fig. 2).

**Table 2:** The characteristics of recurrent cases

Patient	Age/sex	Max tumor size(mm)	ETE	No. of foci	Primary tumor laterality	TNM stage	Sum diameter of all tumors	CLNR	CLNM	Initial operation	Time to recurrence (month)	Site of locoregional recurrence
1	42/F	0.8	+	4	L	I	1.9	1/6	+	HT	5	Contralateral lobe
2	56/M	0.4	−	2	L	III	0.8	3/5	+	HT	78	Contralateral lobe
3	67/F	0.5	−	4	L	III	1.8	1/6	+	HT	25	Contralateral lobe
4	34/M	0.5	−	2	L	I	0.7	1/6	+	HT	92	Contralateral lobe
5	37/M	0.7	−	3	R	I	1.7	0/5	−	HT	35	Contralateral lobe
6	71/F	0.5	−	2	L	III	0.9	4/5	+	HT	37	Ipsilateral central
7	65/F	0.4	−	3	L	I	1.1	0/4	−	HT	48	Contralateral lobe
8	43/M	0.3	−	3	R	I	0.9	1/5	+	HT	63	Contralateral lobe
9	47/M	0.9	−	2	L	III	1.6	1/8	+	HT	66	Contralateral lobe
10	48/F	0.4	−	2	R	III	0.7	3/7	+	HT	73	Contralateral lobe
11	51/M	0.4	−	5	R	III	1.9	3/5	+	HT	83	Contralateral lobe
12	52/F	0.9	+	2	R	III	1.3	2/6	+	HT	89	Contralateral lobe
13	24/M	0.8	−	4	R	I	2.2	2/9	+	HT	103	Contralateral lobe
14	39/F	0.8	+	3	R	I	1.1	3/11	+	HT	91	Contralateral lobe
15	65/M	0.3	−	2	R	III	0.6	1/8	+	HT	67	Contralateral lobe
16	48/F	0.5	+	3	L	IV	1.4	1/11	+	TT	81	Contralateral central
17	66/F	0.5	+	3	R	IV	1.4	2/4	+	TT	102	Ipsilateral lateral

*ETE* extrathyroid extension, *CLNR* central lymph node ratio, *CLNM* central lymph node metastasis

There were huge concerns about that most patients with UMPTMC may have microfoci of PTC in the contralateral lobe. They may believe that recurrent nodule is not newly occurred in the contralateral lobe but microfoci which not be detected at the time of diagnosis. We compared the characteristics of contralateral lobe between HT and TT groups. Fourteen recurrent cases were PTMC which is diagnosed by FNAC in HT group; meanwhile, only two cases had microfoci of PTC in the contralateral lobe in TT group at the time of diagnosis. *p* value was less than 0.05 (*p* = 0.037), and the difference was significant. Furthermore, we also analyzed the clinicopathological features in the contralateral lobe between HT and TT. The differences were not significant maybe owing to the small number of patients (data shown in Table 3).

**Table 3:** Clinicopathological variable of contralateral lobe

Characteristics	Contralateral lobe		
	HT (*n* = 57)	TT (*n* = 40)	*p value*
PTMC			
Yes	14	2	
Tumor size (mm) mean ± SD	0.75 ± 0.23	0.21 ± 0.02	0.085
Microscopic ETE (+)	2	0	0.510
Multifocal (+)	3	0	0.265
With chronic thyroiditis (+)	1	0	1.000
No	42	38	*0.037* ^a^
Benign nodule			
Yes	7	10	
No	50	30	0.105

*SD* standard deviation, *PTMC* papillary thyroid microcarcinoma, *ETE* extrathyroidal extension

^a^Comparison on the PTMC rate in the contralateral lobe between HT and TT

Univariate analysis by Cox’s proportional hazards method showed male gender, sum diameter of all tumors >1 cm, and CLNM to be risk factors for recurrence of HT patients (Table 4). Age, extrathyroidal extension, diameter of the largest tumor, foci number, primary tumor laterality, TNM stage, and presence of chronic thyroiditis were not predictors of recurrence. The risk ratio for male gender, sum diameter >1 cm, and positive CLNM were 5.676 [confidence interval (CI) 2.034–15.839; *p* = 0.001], 9.072 [CI 2.533–32.491; *p* = 0.001], and 4.951 [CI 1.114–21.998; *p* = 0.036] in the HT group. No other risk ratios obtained were of interest. Male gender and sum diameter >1 cm were factors identified for multivariate analysis by Cox’s proportional hazards method (Table 5) which yielded risk ratios of 3.037 [CI 1.026–8.988; *p* = 0.045] and 5.475 [CI 1.389–21.572; *p* = 0.015] in the HT group. Accordingly, male gender and sum diameter >1 cm were shown to be independent predictors of disease-free survival.

**Table 4:** Univariate analysis by Cox’s proportional hazards method for disease-free survival

Factors analyzed	HT patients	
	Risk ratio(CI)	*p value*
Age (years)	1.112(0.395–3.130)	0.840
Male gender	5.676(2.034–15.839)	*0.001*
ETE	0.041(0.000–40.879)	0.365
Diameter of the largest tumor	1.688(0.601–4.743)	0.321
Sum diameter of all tumors	9.072(2.533–32.491)	*0.001*
Foci number	2.191(0.697–6.886)	0.179
Primary tumor laterality	0.889(0.322–2.452)	0.820
TNM stage	1.131(0.386–3.312)	0.822
With chronic thyroiditis	1.638(0.369–7.261)	0.516
CLNM	4.951(1.114–21.998)	*0.036*

*ETE* extrathyroidal extension, *CLNM* central lymph node metastases

**Table 5:** Multivariate analysis by Cox’s proportional hazards method for disease-free survival

Factors analyzed	HT patients	
	Risk ratio (CI)	*p value*
Male gender	3.037(1.026–8.988)	*0.045*
Sum diameter of all tumors	5.475(1.389–21.572)	*0.015*
CLNM	2.205(0.446–10.912)	0.332

*CLNM* central lymph node metastases

## Discussion

Many studies have found that multifocal PTC comprised a more aggressive form of PTC since it was associated with more frequent N1a/N1b disease and occurs more frequently in T3/T4 patients [10]. TT was recommended to multifocal PTC patients for the reason multifocality was associated with an increased risk of disease recurrence or persistence [11, 12]. Nevertheless, PTMC was an inert form of PTC with low risk of recurrence and good prognosis. Therefore, how to manage the patients with UMPTMC remains controversial until now.

Therefore, we investigate clinical and pathologic characteristics of these two group patients, who received HT and TT, respectively. Compared between two groups, we find the disease-free survival period was significantly shorter for the HT patients than for the TT patients (*p =* 0.0059 by the log-rank test). Some researches demonstrated most PTMCs had excellent prognosis, and multifocality did not appear to have significant clinical importance in these tumors [12, 13]. Presence of heterogeneity in clinicopathological features and lack of long-time follow-ups lead to the identical disease-free survival period between unifocal and multifocal PTMC. Consistent with our study, Pyo et al. reported that tumor multifocality was significantly correlated with tumor recurrence in PTMCs (odds ratio, 2.002; 95% confidence interval, 1.475 to 2.719, *p* < 0.001) [14]. With an increased risk of recurrence, HT is not a reasonable operation for UMPTMC. If we recommend TT for all UMPTMCs, the patients would be informed about the need for lifelong thyroid hormone replacement and the risks of recurrent laryngeal nerve injury and hypoparathyroidism. Accordingly, by univariate and multivariate analysis by Cox’s proportional hazards methods, we try to ascertain clinical and pathologic predictors of recurrence in UMPTMC.

In our study, univariate analysis showed male gender, sum diameter of all tumors >1 cm, and CLNM to be risk factors for recurrence of HT patients, as reported in previous studies by Kiriakopoulos et al. [10] and Huang et al. [15], although they focused on multifocal PTC. Kiriakopoulos et al. also found foci number correlates with male gender and lymph node metastases [10]. According to a consensus report of the European Society of Endocrine Surgeons, they recommended bilateral prophylactic CLND for patients with total tumor diameter (sum of the largest diameter of all foci) greater than 1 cm, because the frequency of subclinical CLNM is higher in multifocal PTC. Combining literatures on multifocal PTC and our study, we believe TT should be considered for UMPTMC, especially with male gender and total tumor diameter greater than 1 cm [6].

The gene(s) responsible for multifocal PTC have not yet been identified, and this disorder is now being investigated on a molecular level. Several researches found most of the patients with multifocal PTC had the BRAF^V600E^ mutation in one or more tumor foci and all BRAF^V600E^-positive multifocal PTC showed more aggressive features [16, 17]. Moreover, many studies have investigated multifocal PTC clonality through a number of approaches, including X-chromosome inactivation, *BRAF* mutation, *RET* rearrangements, loss of heterozygosity, or allelic imbalances of distinct cancer foci [17–19]. High rate of independent clonality found in multifocal PTC patients lends support to the notion of field cancerization, whereby carcinogenic agents would affect a wide range of genetic susceptible cells, resulting in their simultaneous transformation [19]. Based on this theory, the contralateral lobe of UMPTMC has higher risk of recurrence attribute to genetic predispositions, environmental exposures, and epidemiological factors. That explains why we recommend TT for UMPTMC, especially with male gender and total tumor diameter greater than 1 cm.

Our study has several limitations. The first limitation is the small number of patients in the study population; due to the incidence of UMPTMC is low, and we want long duration of follow-up, we just summarized patients between October 2005 and October 2006. The second limitation is that until 2011, TSH-suppressive hormonal therapy was applied to postoperative patients and all cases did not receive radioactive iodine ablation. At that time, there was no guideline in China, which we could follow to recommend TSH-suppressive hormonal and radioactive iodine therapy. Finally, we did not use radioiodine whole body scan and PET scan routinely for all the TT patients. This might have some influence on the accuracy of diagnosis of recurrence in TT group, especially distant metastasis. However, the postoperative patients with negative US and thyroglobulin results are less likely to have positive radioiodine and PET scan. Despite these limitations, our study has important implications for UMPTMC management and provides significant information for PTMC guideline formation. Furthermore, to our knowledge, this study is the first report to recommend the optimal surgery strategy for UMPTMC patients.

## Conclusions

Taken together, with an increased risk of recurrence, TT may be more reasonable as initial surgery in UMPTMC, especially with male gender and total tumor diameter greater than 1 cm. A multicentric pragmatic randomized controlled clinical trial with large population is needed to assess the effects on recurrence, quality of life, and cost-effectiveness outcomes of different treatments and follow-up regimen for UMPTMC.

## Additional file

Additional file 1: The ethics statement of our study by the Ethics Committee of Jilin University affiliated First Hospital. (DOC 58 kb)

## Abbreviations

BRAF: B-Raf and v-Raf murine sarcoma viral oncogene homolog B
CLND: Central lymph node dissection
CLNM: Central lymph node metastases
FNAC: Fine needle aspiration cytology
HT: Hemithyroidectomy
PTC: Papillary thyroid carcinoma
PTMC: Papillary thyroid microcarcinoma
RET: Rearranged during transfection
TT: Total thyroidectomy
UMPTMC: Unilateral multifocal PTMC
