# Supplementary material for: Total thyroidectomy may be more reasonable as initial surgery in unilateral multifocal papillary thyroid microcarcinoma: a single-center experience
Source: World J Surg Oncol. 2017 Mar 16;15:62. doi: 10.1186/s12957-017-1130-7 (PMC5356282; doi:10.1186/s12957-017-1130-7)
Supplement: Additional file 1: — The ethics statement of our study by the Ethics Committee of Jilin University affiliated First Hospital. (DOC 58 kb) [file 12957_2017_1130_MOESM1_ESM.doc]

**The University of Jilin**

**The First Hospital of Jilin University**

**Faculty of Medicine**

**Ethics Committee**

**President: Tan Liuquan Committee’s reference number: CR-2016-218**

**Committee member: Sun Jian**

**Niu Junqi**

**Xu Wei**

**Yao Chen**

**Li Wei**

**Application for Approval of Research Protocol**

**1. Title of the Proposed Project**

Central Lymph Node Metastasis as a Predictor for Lateral Lymph Node Metastasis

in Clinically Node-Negative T3 and T4 Papillary Thyroid Carcinoma

**2. Principal and Co-Investigators and Their Departments**

Principal Investigator(s): Guang Chen Professor

The First Hospital/University of Jilin

Co-Investigato(s) Shuai Xue Doctor

The First Hospital/University of Jilin

Jia Liu Associate Professor

The First Hospital/University of Jilin

Peisong Wang Doctor

The First Hospital/University of Jilin

**3. Outline of the Project**

Aim of study :

The objective of the study was to assess the patterns of lateral lymph node metastasis and evaluate the impact of central lymph node metastasis on lymph node resection at level II-V in clinically node-negative（cN0）T3 and T4 PTC patients.

Background :

Papillary thyroid carcinoma is the most common type of thyroid malignancy with a good prognosis [1]. But approximately 40–90% of PTC patients have cervical lymph node metastasis, and much higher in T3 and T4 patients [2]. Cervical lymph node metastasis does not affect the overall prognosis of PTC. However, it enhances the regional recurrence that often needs reoperation with more complications. Therefore, local lymph node recurrence after PTC operation is a major issue that concerns clinicians. Cervical lymph node metastasis is relatively predictable, for thyroid and neck lymphatic drainage patterns are relatively fixed. In the majority of PTC patients, tumors initially spread from the thyroid gland leading to central lymph node metastasis (CLNM). And then lateral lymph nodes are gradually involved [3-4].This phenomenon reflects the echelons of lymphatic drainage.

Current guidelines do not recommend prophylactic lateral lymph node dissection（LLND），because of the low rate of occult metastases, a high accuracy rate of ultrasonographic detection of lateral lymph node metastasis (LLNM) , and the low morbidity associated with secondary surgery in the lateral neck [5].

Prophylactic LLND was performed for the T3 and T4 PTC patients as a means of completely staging the neck to classify patients into risk groups and to determine personalized adjuvant treatment with ^131^I. Using this method, some studies have shown occult metastases in the lateral neck are found in up to 50 % of patients [6-9]. According to these observations, prophylactic LLND may be essential to T3 and T4 PTC patients，though they have no clinically node-positive (ultrasound positive or palpable positive) LLNM. Consequently, a hypothesis is put forward that CLNM may be a good predictor for LLNM in clinically node-negative (cN0) T3 and T4 patients. To this end, the objective of this study was to assess the patterns of LLNM and evaluate the impact of CLNM on LLND at level II–V during neck dissection in T3 and T4 PTC patients.

References :

1. Kepenekci I, Demirkan A, Cakmak A, Tug T, Ekinci C (2009) Axillary Lymph Node Metastasis as a Late Manifestation of Papillary Thyroid Carcinoma. *Thyroid* 19:417-419.

2. Lee BJ, Wang SG, Lee JC, Son SM, Kim IJ, Kim YK (2007) Level IIb lymph node metastasis in neck dissection for papillary thyroid carcinoma. *Arch Otolaryngol Head Neck Surg* 133:1028-1030.

3. Xiao GZ, Gao L (2010) Central Lymph Node Metastasis: Is It a Reliable Indicator of Lateral Node Involvement in Papillary Thyroid Carcinoma? *World J Surg* 34:237-241.

4. Moo TA, Umunna B, Kato M, Butriago D, Kundel A, Lee JA, Zarnegar R, Fahey TJ 3rd (2009) Ipsilateral Versus Bilateral Central Neck Lymph Node Dissection in Papillary Thyroid Carcinoma. *Ann* *Surg* 250:403-408.

5. Dana MH, Abir AG, Isabelle B, Sophie L, Haitham M, Martin S (2014) Prophylactic level II neck dissection guided by frozen section for clinically node-negative papillary thyroid carcinoma: Is It Useful? World J Surg 38:667–672.

6. Ito Y, Tsushima Y, Masuoka H et al (2011) Significance of prophylactic modified radical neck dissection for patients with low-risk papillary thyroid carcinoma measuring 1.1–3.0 cm: first report of a trial at Kuma Hospital. Surg Today 41:1486–1491

7. Machens A, Holzhausen HJ, Dralle H (2004) Skip metastases in thyroid cancer leaping the central lymph node compartment. Arch Surg 139:43–45

8. Mirallie´ E, Visset J, Sagan C et al (1999) Localization of cervical node metastasis of papillary thyroid carcinoma. World J Surg 23:970–973.

9. Hartl D, Leboulleux S, Al Ghuzlan A et al (2012) Optimization of staging of the neck with prophylactic central and lateral neck dissection for papillary thyroid carcinoma. Ann Surg 255: 777–783

Methods :

We performed a retrospective single-center study (2003-2008) of consecutive patients surgically treated at our center for cN0 T3 or T4 PTC with total thyroidectomy and prophylactic bilateral central and ipsilateral lateral neck dissection of ipsilateral levels II, III, IV and V. All patients recruited in the study met the following criteria: a) patient information found in a hospital database and b) patients with a postoperative pathological diagnosis of T3 or T4 PTC. c) patients were performed prophylactic CLND and LLND without clinically positive (Ultrasound positive or palpable positive) central and lateral lymph node preoperatively. Patients were excluded from the study if they had a history of neck radiotherapy or distant metastasis or with history of previous thyroid surgery.

**4. State the questions to be answered, the measurements to be made and how the data will be analysed.**

Clinical diagnosis was initially made by examination of ultrasound and fine needle aspiration, but the results of pathologic examination of the intraoperative frozen sections (FS) were crucial, because they guided the extent of the surgical operation in PTC patients.

The levels of calcium, phosphorus and ionized calcium of all patient were examined preoperatively and also measured routinely at 2 days after surgery. Postoperative hypoparathyroidism is defined as any hypocalcemic symptom with decreased serum calcium level (less than 8.0 mg/dL) or ionized calcium (less than 1.00 mmol/L) level with an elevated phosphorus level (more than 5.0 mg/dL). Permanent hypoparathyroidism is defined as the case that requires medication more than 12 months after surgery. Transient hypoparathyroidism is defined as the absence of hypocalcemic symptoms if the medication was stopped within the 12-month period. Calcium and vitamin D were administered, according to the laboratory findings and hypocalcemic symptom.

Before surgery and on day 1 after surgery, laryngoscopy (either indirect or direct video laryngoscopy) was mandatory. An additional examination was scheduled at 1, 2, 4, 6 and 12 months after surgery in patients with RLN paresis, or until the vocal cord function was recovered. Vocal cord paresis for more than 12 months after surgery was regarded as permanent palsy.

TSH-suppressive hormonal therapy was applied to all postoperative patients and radioactive iodine therapy was used to the case that with gross extrathyroidal extension of the tumor regardless of tumor size, primary tumor size >4 cm or 1–4cm thyroid cancers confined to the thyroid who have documented lymph node metastases, or other higher risk features. Postoperative physical examinations were performed every 3–6 months. During the period of follow-up, all patients underwent ultrasound (US) examinations of the neck as well as thyroid function test. The patients with total thyrodectomy plus bilateral CLND were monitored by the thyroglobulin examination and I-131 scan. We took both the central and lateral compartment into account as the local regional recurrence. A follow-up between 5 and 10 years post-surgery was achieved for all patients.

Statistical analyses were performed using χ^2^ tests. Odds ratios (OR) and 95% confidence intervals (CI) for relationships between each variable and lymph node metastasis (yes or no) were calculated using binary logistic regression. For the non-bivariate normally distributed data, we chose the rank correlation Spearman coefficient and Kendall coefficient methods. SPSS 18.0 software was used for data processing and bilateral inspection; *P* < 0.05 was considered statistically significant.

**5. Specify the number and type of patients or other subjects likely to be involved.**

One hundred and fifty one patients will be recruited into the study. The patients will be recruited as stated above.

**6. State the likely duration of the project and where it will be undertaken.**

A follow-up between 5 and 10 years post-surgery was achieved for all patients. The project will be undertaken at the Department of Thyroid Surgery, the First Hospital of Jilin University.

**7. State the potential hazards to subjects, if any, and the precautions to be taken to meet them.**

As stated above, the procedures involved no risk for patients.

**8. State the procedures which may cause discomfort and distress to subjects and the degree of discomfort or distress likely to be entailed.**

Again, see points 7.

**9. State the manner in which the subject's consent will be obtained.**

We obtain the subject's consent by information consent form.

**10. Any other relevant matters: for instance, letters or information sheets to subjects, payments to subjects, copies of advertisements for volunteers, etc.**

No relevant matters.

**11. If the projects involves the use of a drug, appliance or device, state its exact regulatory status. Is the study sponsored or initiated by an industrial company? What arrangements, if any, for compensation in the event of injury to subjects (where there is no fault) have been made?**

No drug, appliance or device is involved in the project. The study is not initiated nor sponsored by a Pharmaceutical Company.

**12. Is there any interest, i.e., of profit, personal or departmental, relating to the study?**

There is no profit derived personally, or by the department.

Corresponding author:

**
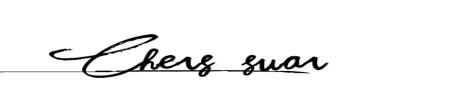

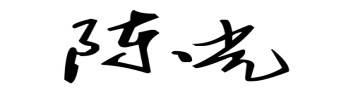

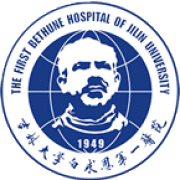
**
